# Evaluation and correlation of patient movement during online adaptive radiotherapy with CBCT and a surface imaging system

**DOI:** 10.1002/acm2.14133

**Published:** 2023-08-29

**Authors:** Dennis N. Stanley, Elizabeth Covington, Joseph Harms, Joel Pogue, Carlos E. Cardenas, Richard A. Popple

**Affiliations:** ^1^ Department of Radiation Oncology University of Alabama at Birmingham Birmingham Alabama USA; ^2^ Department of Radiation Oncology Michigan Medicine Ann Arbor Michigan USA

**Keywords:** IDENTIFY, online adaptive radiotherapy, optical surface imaging, patient movement, SGRT

## Abstract

**Purpose:**

With the clinical implementation of kV‐CBCT‐based daily online‐adaptive radiotherapy, the ability to monitor, quantify, and correct patient movement during adaptive sessions is paramount. With sessions lasting between 20–45 min, the ability to detect and correct for small movements without restarting the entire session is critical to the adaptive workflow and dosimetric outcome. The purpose of this study was to quantify and evaluate the correlation of observed patient movement with machine logs and a surface imaging (SI) system during adaptive radiation therapy.

**Methods:**

Treatment machine logs and SGRT registration data log files for 1972 individual sessions were exported and analyzed. For each session, the calculated shifts from a pre‐delivery position verification CBCT were extracted from the machine logs and compared to the SGRT registration data log files captured during motion monitoring. The SGRT calculated shifts were compared to the reported shifts of the machine logs for comparison for all patients and eight disease site categories.

**Results:**

The average (±STD) net displacement of the SGRT shifts were 2.6 ± 3.4 mm, 2.6 ± 3.5 mm, and 3.0 ± 3.2 in the lateral, longitudinal, and vertical directions, respectively. For the treatment machine logs, the average net displacements in the lateral, longitudinal, and vertical directions were 2.7 ± 3.7 mm, 2.6 ± 3.7 mm, and 3.2 ± 3.6 mm. The average difference (Machine–SGRT) was −0.1 ± 1.8 mm, 0.2 ± 2.1 mm, and −0.5 ± 2.5 mm for the lateral, longitudinal, and vertical directions. On average, a movement of 5.8 ± 5.6 mm and 5.3 ± 4.9 mm was calculated prior to delivery for the CBCT and SGRT systems, respectively. The Pearson correlation coefficient between CBCT and SGRT shifts was *r* = 0.88. The mean and median difference between the treatment machine logs and SGRT log files was less than 1 mm for all sites.

**Conclusion:**

Surface imaging should be used to monitor and quantify patient movement during adaptive radiotherapy.

## 1 INTRODUCTION

With the clinical implementation of kV‐CBCT‐based daily online‐adaptive radiotherapy, the ability to monitor, quantify, and correct patient movement during adaptive sessions is paramount. Online adaptive therapy corrects for daily variations in patient set‐up and anatomy by generating a new radiotherapy treatment plan using daily imaging to update structure contours.^1^, ^2^, ^3^, ^4^, ^5^, ^6^, ^7^ Patients are kept in the treatment position for the entire adaptive session, which typically lasts between 20−45 min, leading to an increased likelihood of patient motion.^7^ Typically, radiographic imaging is repeated prior to treatment to verify the integrity of the patient setup and acceptability of anatomical changes (e.g. bladder filling). Significant changes in patient setup during adaptation may indicate the need to restart the adaptive session, repeating the 20−45‐min session from scratch. Thus, the ability to quickly detect movement is critical to the adaptive workflow and ensures an optimal dosimetric outcome.

Surface imaging (SI) has been routinely used in radiotherapy to assist in initial patient set‐up,^8^, ^9^ enable respiratory gating,^10^ and monitor intrafraction motion.^10^, ^11^, ^12^ Surface imaging systems utilize camera pods at specified locations to ensure visualization of the patient during set‐up and treatment. The Varian Ethos (Varian Medical Systems, Palo Alto, CA) is a 6MV Flattening Filter Free (6X‐FFF) ring‐based linear accelerator capable of performing intensity‐modulated radiation therapy (IMRT) and volumetric modulated arc therapy (VMAT). For bore‐based treatments, such as on an Ethos or MR‐Linac, visualization of the patient presents a unique challenge. SI systems have addressed this challenge by either utilizing camera pod configurations external to the bore or employing in‐bore cameras.^13^, ^14^, ^15^ The IDENTIFY system has SI cameras placed outside the bore for the Ethos configuration.

The purpose of this study was to evaluate the correlation of observed patient movement using treatment machine logs and SI system logs captured during adaptive radiation therapy. While SI systems are routinely used for monitoring patient motion, there is a need to determine if their accuracy is sufficient for patient repositioning during treatment, especially in the context of bore‐based treatments with non‐standard camera configurations and adaptive treatments with inherently longer treatment times. This study will report on the agreement of SI‐reported intrafraction motion with radiographically determined shifts to determine the feasibility of utilizing SI for correcting intrafraction motion during adaptive radiotherapy.

## 2 METHODS AND MATERIALS

### 2.1 Varian identify and ethos

The Varian IDENTIFY (Varian Medical Systems) is an optical surface imaging (SI) system that consists of three stereoscopic camera pods that project a speckle pattern which is then used to reconstruct camera images into a 3D surface. Similar to other SI systems, Ethos IDENTIFY consists of three ceiling‐mounted camera pods, each containing two cameras and one projector. With respect to a head‐first supine patient, two cameras are Inferior to the gantry and one camera is superior to the gantry. The IDENTIFY is used to monitor inter‐fractional and intra‐fractional motion for all patients and uses a reference surface captured at the time of treatment for positional alignment and motion management throughout treatment.^16^ A region of interest (ROI), defined according to departmental site‐specific guidelines, is selected for monitoring. Its position is compared with the reference surface during treatment to monitor the patient's overall movement. The reference image can be either the body structure defined by the planning CT or a generated surface taken during the initial CBCT on the first day. For this study, all the evaluated reference images were acquired during the initial planning CBCT. A new reference surface was captured after the pre‐delivery CBCT if the patient's position was adjusted. Differences between the reference surface and a live image, including longitudinal, lateral, vertical, and total magnitude, are monitored during setup and delivery. During treatment, the patient's position is recorded in the registration data log files.

The Varian Ethos has a treatment gantry speed of up to four rotations per minute (4 RPM) and a dual‐stacked, 28 cm^2^ × 28 cm^2^ at maximum, multileaf collimator with maximum leaf speeds of 5.0 cm/s. The Ethos is equipped with MV and kV imagers inside of an enclosed 100 cm wide bore. The Ethos has a magnetically driven couch with a 226 kg weight limit, and 41.6 cm, 47.5 cm, and 165.5 cm of travel in the lateral, vertical, and longitudinal directions, respectively. For the Ethos, only the kV‐CBCT is usable during clinical operation.

### 2.2 Data collection and analysis

In this IRB‐approved (IRB‐120703005) retrospective study, Ethos machine logs and IDENTIFY registration data log files from 1972 individual adaptive sessions for 117 patients treated between August 2021 and December 2022 at our institution were exported and analyzed. Patients were positioned and immobilized according to our institutions’ standard site‐specific clinical procedure utilizing Vac‐Lok immobilization cushions, Alpha Cradle molds, or open‐face masks. The applied shifts from a pre‐delivery positional verification CBCT were extracted from the Ethos machine logs and compared to the IDENTIFY registration data log files captured during motion monitoring for each session. Adjustments to patient positioning were only applied in conjunction with radiographic imaging. For the Ethos machine logs, the applied shift of the verification CBCT was recorded, verified using the treatment timestamps and set as the ground truth. For the IDENTIFY log files, the shift ($P_{shift\_SI})$ was calculated by taking the absolute difference between the average position five seconds immediately pre‐shift (preSI) and post‐shift (postSI) in each direction, shown in Equation (1).

(1) $$P_{shift\_SI}=\sqrt{{\left(Vrt_{postSI}-Vrt_{preSI}\right)}^{2}+{\left(Lng_{postSI}-Lng_{preSI}\right)}^{2}+{\left(Lat_{postSI}-Lat_{preSI}\right)}^{2}}$$

The Ethos machine log files and IDENTIFY registration log files were then matched based on the treatment timestamp. Timestamp congruence between IDENTIFY and Ethos was independently verified by benchmarking against the completion of the daily QA. The magnitude of patient movement reported by IDENTIFY, *P*_mag_, was calculated by adding the three Cartesian coordinate movements in quadrature from the IDENTIFY pre‐shift values designated by the timestamps acquired from the treatment machine log files as shown in Equation (2).

(2) $$P_{mag}=\sqrt{Vrt_{preSI}^{2}+Lng_{preSI}^{2}+Lat_{preSI}^{2}}$$

Since the initial timepoint value is always 0,0,0 in IDENTIFY, this represents the total intrafraction movement detected by IDENITFY prior to the application of the shift. *P*_shift_Ethos_ was calculated utilizing the absolute difference of table positions as seen in Equation (3).

(3) $$P_{shift\_Ethos}=\sqrt{{\left(Vrt_{post}-Vrt_{pre}\right)}^{2}+{\left(Lng_{post}-Lng_{pre}\right)}^{2}+{\left(Lat_{post}-Lat_{pre}\right)}^{2}}$$

The IDENTIFY calculated shifts $P_{shift\_SI}$ were compared and correlated to the reported shifts of the machine logs $P_{shift\_Ethos}$. Data processing and correlation were done in MATLAB (Mathworks, Natick, MA). Additionally, a sub‐analysis was performed to determine if patient‐specific factors like treated disease site, skin tone, or time on the table led to differences between the surface imaging and CBCT‐calculated shifts. A Mann‐Whitney‐U two‐sided test was used with a significance threshold of *p* = 0.05.

## 3 RESULTS

**FIGURE 1:**
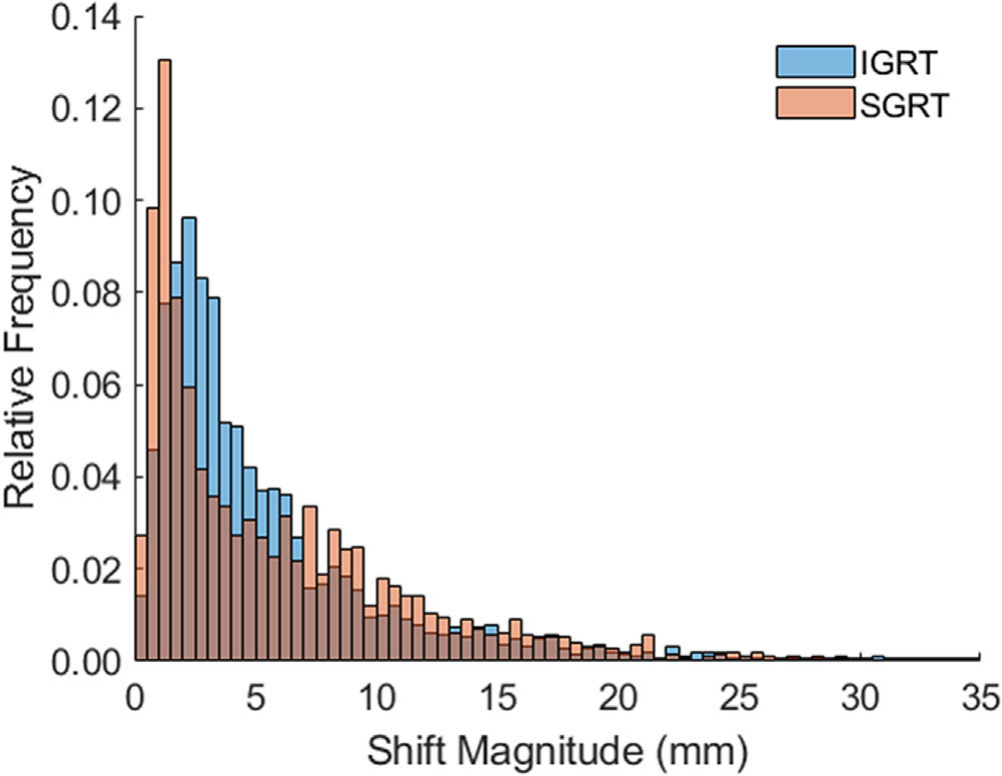
The magnitude of the detected patient movements for Ethos Machine logs and IDENTIFY registration data logs. The average (± STD) net displacement of the IDENTIFY shifts were 2.6 ± 3.4 mm, 2.6 ± 3.5 mm, and 3.0 ± 3.2 mm in the lateral, longitudinal, and vertical directions respectively. For the Ethos machine logs, the net displacements in the lateral, longitudinal, and vertical directions were 2.7 ± 3.7 mm, 2.6 ± 3.7 mm, and 3.2 ± 3.6 mm, respectively.

**FIGURE 2:**
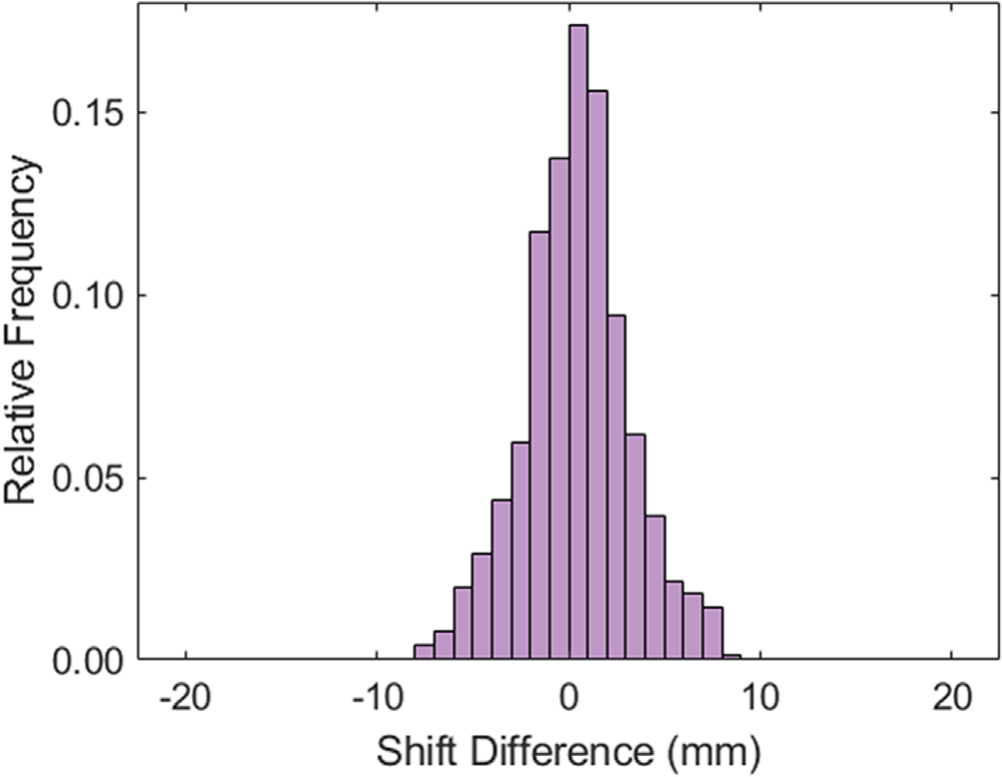
Histogram of difference in the calculated magnitude of the shift between Ethos and IDENTIFY. The average difference in shift magnitude (Ethos‐IDENTIFY) was −0.1 ± 1.8 mm, 0.2 ± 2.1 mm, and −0.5 ± 2.5 mm, for the lateral, longitudinal, and vertical directions, respectively.

**FIGURE 3:**
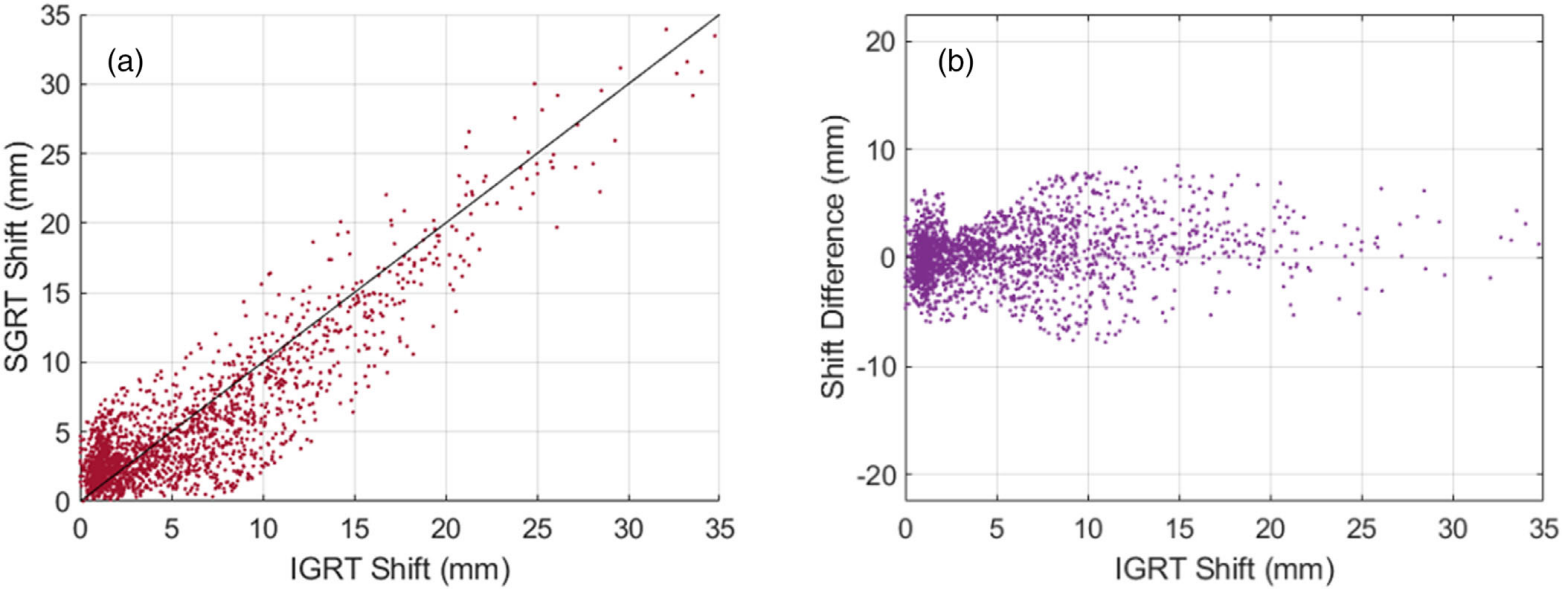
(a) Scatterplot of SGRT shift versus IGRT shift; a Pearson correlation coefficient of *r* = 0.88 was calculated between both sets of data, demonstrating a very high degree of correlation. (b) Shift difference between IGRT and SGRT systems as a function of IGRT shift.

Table 1 shows a summary of the calculated patient movement, the amplitude of shifts for both systems, and the difference between each vector. Figure 1 shows the magnitude of the detected patient movements for Ethos Machine logs and IDENTIFY registration data logs. The average (± STD) net displacement of the IDENTIFY shifts were 2.6 ± 3.4 mm, 2.6 ± 3.5 mm, and 3.0 ± 3.2 mm in the lateral, longitudinal, and vertical directions, respectively. For the Ethos machine logs, the net displacement in the lateral, longitudinal, and vertical directions was 2.7 ± 3.7 mm, 2.6 ± 3.7 mm, and 3.2 ± 3.6 mm, respectively. On average, a movement of 5.8 ± 5.6 mm and 5.3 ± 4.9 mm was calculated prior to delivery for the CBCT and SGRT systems, respectively. Figure 2 shows a histogram of the difference in the calculated magnitude of the shift between Ethos and IDENTIFY. The average difference in shift magnitude (Ethos‐IDENTIFY) was −0.1 ± 1.8 mm, 0.2 ± 2.1 mm, and −0.5 ± 2.5 mm, for the lateral, longitudinal, and vertical directions, respectively. The skewness of the shift difference distribution is 0.0327, indicating that the difference between the SGRT and IGRT systems is highly symmetric and normal in nature. Figure 3a shows a scatterplot of SGRT shift versus IGRT shift; a Pearson correlation coefficient of *r* = 0.88 was calculated between both sets of data, demonstrating a very high degree of correlation. Figure 3b shows shift difference as a function of IGRT shift; there is no clear trend in the difference between both systems as a function of IGRT shift magnitude.

**TABLE 1:** Summary of the calculated absolute displacements and the amplitude of shifts for ethos machine logs and IDENTIFY registration data logs. IQR, interquartile range.

		Ethos machine logs	IDENTIFY registration data log
		(Ave ± STD) [IQR]	(Ave ± STD) [ IQR]
		(mm)	(mm)
**Net displacement**			
	Lateral	2.7 ± 3.7 [ −1.2,1.2]	2.6 ± 3.4 [ −0.9,1.1]
	Longitudinal	2.6 ± 3.7 [−0.7,1.6]	2.6 ± 3.5 [−1.1,1.7]
	Vertical	3.2 ± 3.6 [−1.1,1.8]	3.0 ± 3.2 [−0.4,2.6]
	$P_{shift\_Ethos}$	5.9 ± 5.6 [ 1.4,8.5]	
	$P_{shift\_SI}$		5.4 ± 5.0 [2.2,6.7]
**Calculated patient movement**	$P_{mag}$		5.3 ± 4.9[2.1,6.7]

**Relative difference**		Δ Difference $\left(\;P_{shift_{Ethos}}-\;P_{shift_{SI}}\right)$	IQR
	Δ Lateral	−0.1 ± 1.8	[−0.7,0.6]
	Δ Longitudinal	0.2 ± 2.1	[−0.7,1.0]
	Δ Vertical	−0.5 ± 2.5	[−1.8,0.5]
	Magnitude	0.4 ± 2.7	[−1.2,2.0]
**Pearson correlation coefficient** (*ρ*)		0.88	

**FIGURE 4:**
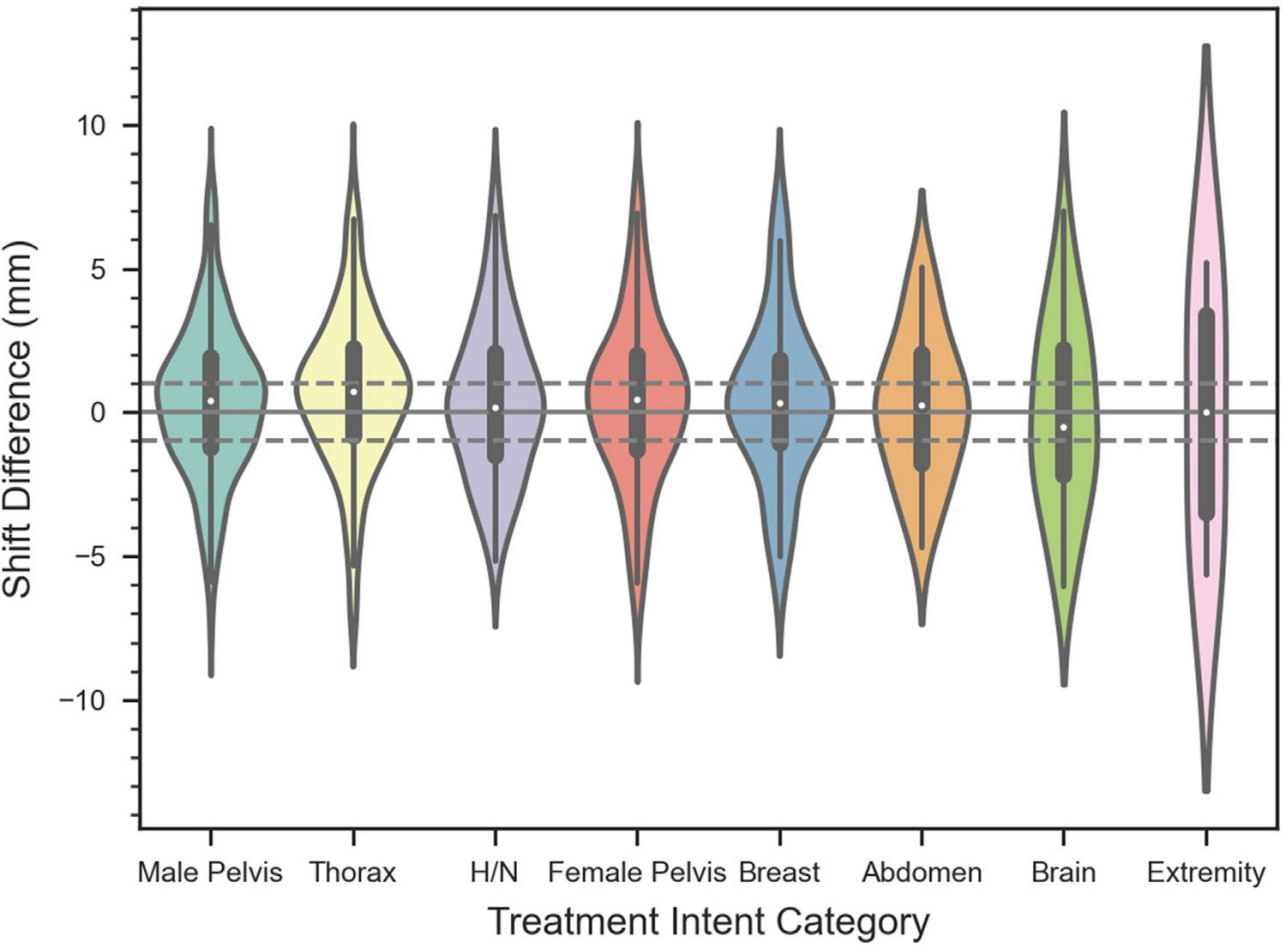
Violin plot of the shift difference between the IGRT and SGRT systems as a function of treatment site. The White circle shows the median, the thick gray line shows the interquartile range, and the thin gray line shows non‐outlier data.

**FIGURE 5:**
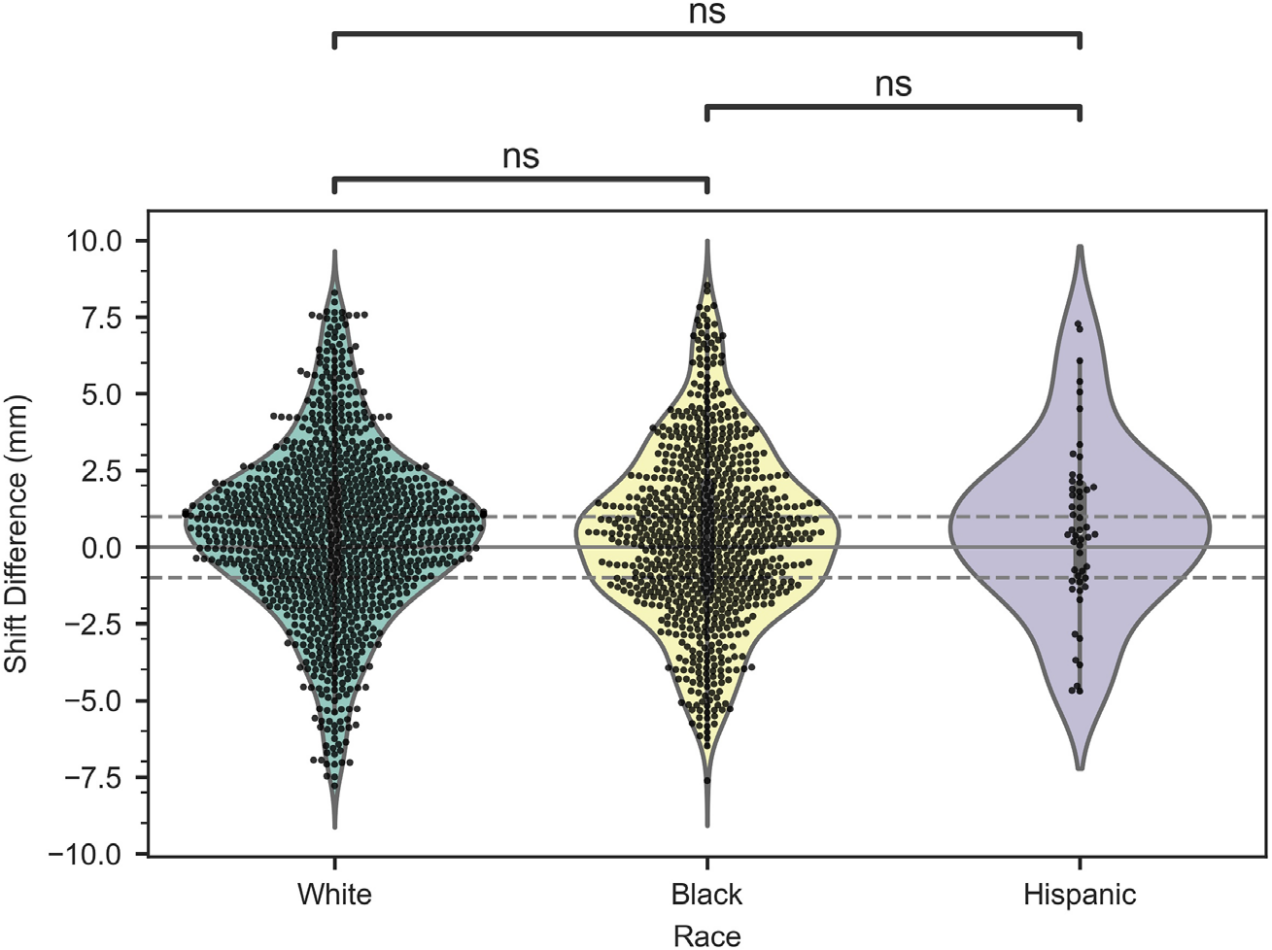
Violin/swarm plot of the shift difference between the IGRT and SGRT systems as a function of race (self‐reported). The thick gray line shows the interquartile range, and the thin gray line shows non‐outlier data. Black dots show individual session data points.

**FIGURE 6:**
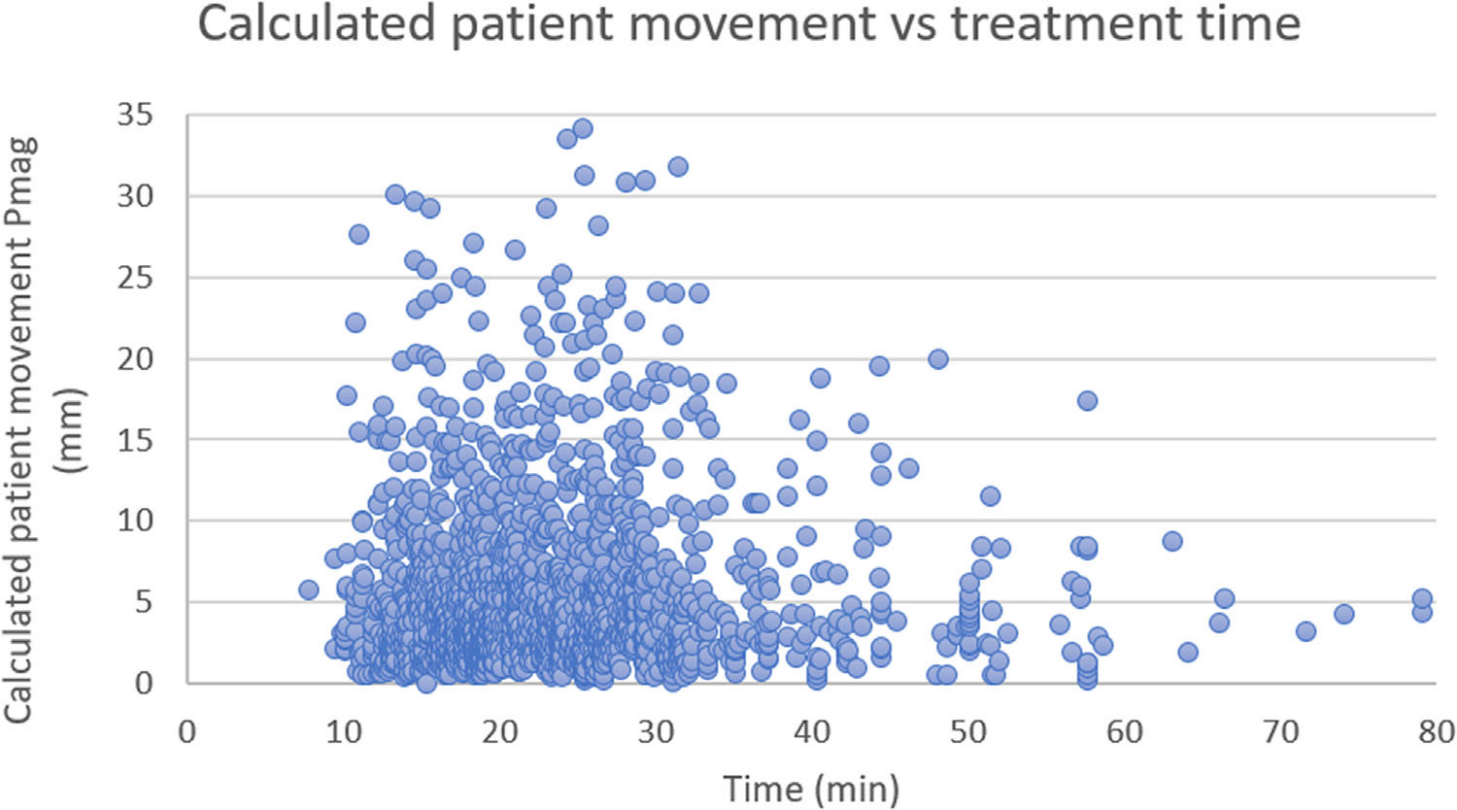
Scatterplot of the IDENTIFY reported patient movement, *P*_mag_, versus time between initial and pre‐delivery CBCTs; a Pearson correlation coefficient of *r* = 0.12 was calculated between both sets of data, demonstrating a very low degree of correlation.

Table 2 shows the average (±STD) Ethos and IDENTIFY machine log shifts and the difference between shifts as a function of the treatment site. It can be seen that the mean difference for the male pelvis, head and neck, female pelvis, and breast are identical (0.4 mm). The thorax shows an increased mean difference (0.7 mm) and the abdomen, brain, and extremity show decreased mean differences (0.2, −0.1, and −0.1 mm, respectively) relative to the other sites. Figure 4 shows a violin plot of the difference between the IGRT and SGRT systems as a function of body site. The median difference for every site is within ±1 mm. Only the brain has a negative mean and median difference. The extremities show the largest spread, but there are only 4 evaluable cases. The abdomen, brain, and extremity sites are composed of 25, 28, and 4 sessions; thus, inferences drawn for these sites are not statistically viable without more data. The difference between systems as a function of self‐reported race is shown in Table 3 and Figure 5. The mean difference (± STD) for the Black, White, and Hispanic patients were 0.4 ± 2.8 mm, 0.5 ± 2.7 mm, and 0.6 ± 2.8 mm respectively. A Mann‐Whitney‐Wilcoxon two‐sided test was performed between each of the race cohorts with p‐values greater than 0.05 for each comparison (shown in Figure 5). Figure 6 shows a scatterplot of IDENTIFY reported movement (P_mag_) versus time between the initial and pre‐delivery CBCT; a Pearson correlation coefficient of *r* = 0.12 was calculated between both sets of data, showing that more time on the treatment couch did not correlate with more patient motion.

**TABLE 2:** Summary of the calculated displacement magnitudes and difference between the IGRT and SGRT systems for each treatment site.

	Ethos machine logs $P_{shift\_Ethos}$	IDENTIFY registration data log $P_{shift\_SI}$	Δ Difference $\left(\;P_{shift_{Ethos}}-\;P_{shift_{SI}}\right)$
**Treatment Site**	Ave ± STD (mm)	Ave ± STD (mm)	Ave ± STD (mm)
Male Pelvis	5.3 ± 5.2	4.9 ± 4.5	0.4 ± 2.7
Thorax	6.9 ± 7.2	6.3 ± 6.7	0.7 ± 2.7
H/N	5.8 ± 4.9	5.7 ± 4.0	0.4 ± 2.7
Female Pelvis	6.6 ± 6.0	6.1 ± 5.5	0.4 ± 2.9
Breast	5.5 ± 4.4	5.1 ± 3.7	0.4 ± 2.9
Abdomen	4.8 ± 3.4	4.4 ± 2.3	0.2 ± 2.5
Brain	5.7 ± 4.2	5.4 ± 2.1	−0.1 ± 3.3
Extremity	7.5 ± 5.3	5.9 ± 2.1	−0.1 ± 5.0

**TABLE 3:** Summary of the calculated displacement magnitudes and difference between the IGRT and SGRT systems for each race.

	Ethos machine logs $P_{shift\_Ethos}$	IDENTIFY registration data log $P_{shift\_SI}$	Δ Difference $\left(\;P_{shift_{Ethos}}-\;P_{shift_{SI}}\right)$
**Race (self‐reported)**	Ave ± STD (mm)	Ave ± STD (mm)	Ave ± STD (mm)
White	5.6 ± 5.3	5.1 ± 4.8	0.5 ± 2.7
Black	6.2 ± 6.0	5.9 ± 5.4	0.4 ± 2.8
Hispanic	4.9 ± 5.5	4.5 ± 5.2	0.6 ± 2.8

## 4 DISCUSSION

perceived benefit of adaptive therapy is the ability to reduce treatment margins as daily variations in set‐up and anatomical changes can be addressed by replanning.^17^ In this study, both SI and radiographic imaging reported an average magnitude of translational motion greater than 4 mm for all sites, indicating the need to monitor and correct for intrafraction motion during the adaptive session. According to Stanley et al “average(±std), adaptive sessions took 34.52 ± 11.42 min from start to finish. The entire adaptive process (from the start of contour generation to verification CBCT), performed by the physicist (and physician on select days), was 19.84 ± 8.21 min.” Figure 2 shows that the average magnitude of translational intrafraction motion can exceed margins typically utilized in adaptive therapy. A difference in the mean magnitude of CBCT and IDENTIFY of 0.4 ± 2.7 mm indicates that SI is adept at capturing intrafraction motion and informing the treatment team that evaluation of patient set‐up is needed.

While the absolute difference between IDENTIFY SI logs and shifts recorded from radiographic imaging on average were submillimeter in each translational axis, the larger standard deviations require further investigation. Some of the variation potentially arises because the external surface is not a perfect surrogate for internal target motion, leading to a discrepancy between the IGRT and SGRT systems. More explicitly, differences between IGRT and SGRT systems may arise because soft tissue targets used to align the adaptive plan move either more or less than patient surfaces. As an example, the prostate bed may experience different vector movement than the abdominal skin centroid during the time between initial and verification CBCT due to its location between the bladder and rectum.

There are also other patient‐specific attributes, like skin tone or body habitus, that may reduce the accuracy of the SGRT‐reported offsets. Previous work has shown inferior performance of SI systems with patients with darker skin tones that led to significant differences in SI performance with non‐zero couch positions and during camera occlusion.^18^ In the current study, we investigated SI performance utilizing self‐reported race as a surrogate for skin tone. Since the Ethos system does not utilize couch rotations and cannot be obscured by gantry motion, any difference observed was expected to be a function of suboptimal camera settings to equally visualize all skin tones. Task Group 302^19^ also recommends that SI systems should be tested to ensure that camera settings are appropriate for a variety of skin tones. Due to a lack of SI phantoms covering the spectrum of skin tones represented in our patient population, we utilized clinical data to elucidate any differences in performance. Future studies include studying additional patient‐specific factors that may contribute to the differences observed.

The data collected in the study is indicative of one SI system and may not be representative of all systems due to variations in camera set‐up, the camera performance of other systems, and differences in commissioning and calibration methods. Clinics utilizing SI should evaluate their system's performance and establish data‐driven guidelines for utilizing SI and setting thresholds for repeating radiographic imaging. Overall, SI is adept at capturing patient motion but may not have the perceived accuracy for patient repositioning; therefore, we recommend maintaining the use of radiographic imaging as the gold standard for verification of patient set‐up during adaptive therapy.

## 5 CONCLUSIONS

Surface imaging should be used to monitor and quantify patient movement during adaptive radiotherapy for many sites. However, it does not replace the need for a pre‐delivery verification CBCT, especially in clinical sites prone to anatomical changes during the adaptive process.

## AUTHOR CONTRIBUTIONS

All authors listed above made substantial contributions to the conception and design of the work; the acquisition, analysis, and interpretation of data for the work; drafting the work and revising it critically for important intellectual content; gave final approval of the version to be published; and agrees to be accountable for all aspects of the work in ensuring that questions related to the accuracy or integrity of any part of the work are appropriately investigated and resolved.

## CONFLICT OF INTEREST STATEMENT

Dennis N. Stanley, Ph.D.—Dennis Stanley has received research support, not related to this work, and speaker honoraria from Varian medical systems Joseph Harms, Ph.D.—Nothing to disclose. Joel A. Pogue, Ph.D.—Nothing to disclose. Richard A Popple, Ph.D.—Richard Popple has received research support, not related to this work, and speaker honoraria from Varian medical systems Carlos E. Cardenas, Ph.D.—Nothing to disclose.
